# Benchmarking of Hi-C tools for scaffolding plant genomes obtained from PacBio HiFi and ONT reads

**DOI:** 10.3389/fbinf.2024.1462923

**Published:** 2024-11-15

**Authors:** Lia Obinu, Urmi Trivedi, Andrea Porceddu

**Affiliations:** ^1^ Department of Agricultural Sciences, University of Sassari, Sassari, Sardinia, Italy; ^2^ Edinburgh Genomics, The University of Edinburgh, Edinburgh, United Kingdom

**Keywords:** genome scaffolding, Hi-C reads, *de novo* genome assembly, assemblyQC, benchmarking

## Abstract

The implementation of Hi-C reads in the *de novo* genome assembly process allows the ordering of large regions of the genome in scaffolds and the generation of chromosome-level assemblies. Several bioinformatics tools have been developed for genome scaffolding with Hi-C, and each tool has advantages and disadvantages that need to be carefully evaluated before their adoption. We generated two *de novo* assemblies of *Arabidopsis thaliana* obtained from the same raw PacBio HiFi and Oxford Nanopore Technologies data. We scaffolded the assemblies implementing Hi-C reads with the scaffolders 3D-DNA, SALSA2, and YaHS, with the aim of identifying the tool providing the most accurate assembly. The scaffolded assemblies were evaluated according to contiguity, completeness, accuracy, and structural correctness. In our analysis, YaHS proved to be the best-performing bioinformatics tool for scaffolding *de novo* genome assemblies in *Arabidopsis thaliana*.

## 1 Introduction

Third-generation sequencing (TGS) technologies, for example, Pacific Biosciences (PacBio) and Oxford Nanopore Technologies (ONT), produce long reads that can be used to obtain high-quality genome sequences. Compared to next-generation sequencing technologies, TGS provides better resolution and contiguity, allowing partial resolution of the assembly of repeats and duplications, which is crucial for resolving repeat-rich genomic regions such as centromeres and telomeres, especially in plant genomes (Jung et al., 2019).

Although long read assembly alone can yield long contigs, this process can still be far from assembling at the chromosome scale without additional scaffolding efforts. Strategically, larger genome projects such as the Vertebrate Genome Project (VGP–https://vertebrategenomesproject.org/) and the Darwin Tree of Life (DToL–https://www.darwintreeoflife.org/) combine multiple data types such as optical mapping (Zhou et al., 2007) and Hi-C (Burton et al., 2013) to construct reliable chromosome-scale assemblies (Guan et al., 2021). Hi-C reads are also part of the standards and recommendations for genome assembly of the Earth BioGenome Project (Lawniczak et al., 2022).

Originally developed to study the three-dimensional organization of the genome (Lieberman-aiden et al., 2009), Hi-C reads are now largely employed for scaffolding *de novo* genome assemblies (Ghurye and Pop, 2019). Hi-C technology is a method that combines proximity-based ligation with massively parallel sequencing, allowing the unbiased identification of chromatin interactions across an entire genome. This allows grouping, ordering, and orienting contigs (i.e., contiguous sequences) based on chromatin contact frequency between different genomic regions (Yamaguchi et al., 2021), resulting in accurate chromosome-level assemblies (Marie-Nelly et al., 2014).

High-quality, chromosome-level genome assembly is essential in plant science and genetic research, as it influences our comprehension of the genetic architecture of significant traits and facilitates marker selection and candidate gene identification (Benevenuto et al., 2019). In addition, it enables comparative analysis of structural features and gives insights into the evolutionary history of plant genomes (Shirasawa et al., 2021). Hi-C is currently the favored method of *de novo* genome scaffolding because, unlike optical mapping, it does not necessarily require the extraction of super-long genomic DNA fragments (Shirasawa et al., 2021). In addition, optical mapping methodologies are technically demanding and necessitate optimization for each species (Michael and VanBuren, 2020). Hi-C reads enhance the differentiation of contigs from distinct chromosomes and ensure the continuity of scaffolding outcomes (Luo et al., 2021). Nonetheless, although Hi-C usually facilitates the resolution of genomes into chromosomes, they may not be sufficient with more complicated plant genomes (Michael and VanBuren, 2020).

The choice of the Hi-C scaffolding bioinformatic tool is crucial for obtaining optimal results in *de novo* assembly, and, therefore, comparative analyses are needed.

A previous benchmarking study of Hi-C-based scaffolders (Sur et al., 2022) evaluated the performance of five different tools: Lachesis (Burton et al., 2013), HiRise (https://github.com/DovetailGenomics/HiRise_July2015_GR), 3d-dna (Dudchenko et al., 2017), SALSA (Ghurye et al., 2017), and AllHiC (Zhang et al., 2019). The study was based on partitioned reference genomes and *de novo* assemblies generated from PacBio CLR reads with Canu (Koren et al., 2017). The scaffolders’ performance evaluation was based on the mapping of assemblies against the known reference genome using MUMmer4 (Marçais et al., 2018) and on a set of accuracy metrics calculated using the Python package Edison (https://github.com/Noble-Lab/edison), which examines scaffolding outcomes in comparison to a reference genome. The assembly evaluation method of this study strongly relied on the existence of a reference genome, leading to a reference-biased assessment that might not be the optimal strategy for *de novo* assembly.

A second benchmarking of Hi-C scaffolders was conducted by Hou et al., 2023. The authors generated several *de novo* assemblies from PacBio HiFi data of different plant species and performed the scaffolding using only simulated Hi-C data. They evaluated the assemblies scaffolded with LACHESIS (Burton et al., 2013), Pin_hic (Guan et al., 2021), YaHS (Zhou et al., 2023), SALSA2 (Ghurye et al., 2019), 3d-DNA (Dudchenko et al., 2017), and ALLHiC (Zhang et al., 2019) based on distinct k-mers using a variety of metrics.

In this study, we benchmarked the three most frequently used Hi-C scaffolders [3D-DNA (Dudchenko et al., 2017), SALSA2 (Ghurye et al., 2019), and YaHS (Zhou et al., 2023)], with the aim of identifying the tool producing the most accurate chromosome-scale *de novo* genome assembly. 3D-DNA was chosen due to its widespread application and ongoing active development. SALSA2 is the new version of SALSA, while YaHS is the most recently released scaffolder. We excluded several tools: LACHESIS (Burton et al., 2013) because it is no longer under development; HiRise (https://github.com/DovetailGenomics/HiRise_July2015_GR, which is not open source; SALSA (Ghurye et al., 2017), which was replaced by SALSA2; ALLHiC (Zhang et al., 2019), which is specifically developed for autopolyploid genomes; and Pin_hic (Guan et al., 2021), which is not extensively used among the community.

We generated two *de novo* assemblies of *A. thaliana*, the model species for plant genomics (Koornneef and Meinke, 2010), using the same raw reads obtained from the BioProject PRJCA005809 (Wang et al., 2022). The dataset was selected for being a plant dataset with an extensive array of reads including PacBio HiFi, ONT, Hi-C, and Illumina. The assemblies were produced using different assembly methodologies to obtain the primary contigs, with the goal of assessing potential discrepancies in the scaffolding results attributable to the assembly strategy.

To facilitate the benchmarking work, we developed assemblyQC, a Bash pipeline that combines QUAST (Mikheenko et al., 2018), BUSCO (Manni et al., 2021), and Merqury (Rhie et al., 2020). These three tools assess the quality of *de novo* assemblies without relying on a reference genome. The pipeline also optionally uses Liftoff (Shumate and Salzberg, 2021) to annotate the *de novo* assembly, and a Python script subsequently analyzes the positioning of genes on the target assembly compared to the reference genome. AssemblyQC automatically launches these tools with minimal user input. It is available on GitHub at https://github.com/LiaOb21/assemblyQC.

## 2 Materials and methods

### 2.1 The *A. thaliana* genome and raw data

The first *A. thaliana* genomic sequence was obtained in 2000 from the Columbia genotype using the minimum tiling path of BACs sequenced with Sanger technology (Kaul et al., 2000). The genome sequence spans approximately 135 Mb in length and is organized into five chromosomes (2n = 10).

In this study, we used publicly available raw data from BioProject PRJCA005809 (Wang et al., 2022) to construct two *de novo* assemblies and evaluate the performance of the three scaffolders and possible inconsistencies in outcomes due to the assembly method.

Table 1 summarizes the characteristics of the raw reads used. Illumina reads were not used for the assembly process, but they were used with Merqury (Rhie et al., 2020) for scaffolding evaluation purposes.

**TABLE 1:** Overview of the characteristics of the raw reads from BioProject PRJCA005809 (Wang et al., 2022) used in the assembly process.

Reads	Mean read length	Mean read quality	Number of reads	Total bases	Coverage
ONT	18,541.30	11.1	3,064,191.00	56,814,196,989.00	420.84507
PacBio HiFi	15,094.40	31.8	1,517,433.00	22,904,700,074.00	169.66475
Hi-C	150	>30	140,957,500	21,143,625,000	156.6194444
Illumina	150	>30	91,309,542	13,696,431,300	101.4550467

### 2.2 *De novo* assembly

ONT reads were trimmed with NanoFilt (Coster et al., 2018) using the parameter -l 500, which only retained the reads with a minimum length of 500 bp.

The first *de novo* assembly was obtained by assembling ONT reads using Flye (Kolmogorov et al., 2019) in --nano-raw mode with default parameters. To subsequently perform the polishing, the PacBio HiFi reads were mapped to the draft assembly using minimap2 (Li, 2018) in map-hifi mode, and then the polishing was performed with Racon (Vaser et al., 2017). Haplotigs and overlaps in the assembly were removed using Purge _dups (Guan et al., 2020). Contaminants were removed using BlobToolKit (Challis et al., 2020) by applying a filter for GC content equal to 0.4 because Tiara (Karlicki et al., 2022) identified contigs with higher GC content as “unknown.” Hereafter, we will refer to this assembly as “Flye.”

The second *de novo* assembly was obtained by assembling the HiFi and ONT reads together using Hifiasm (Cheng et al., 2021) with the default parameters. Haplotigs and overlaps in the assembly were removed using purge_dups (Guan et al., 2020). Contaminants were removed using BlobToolKit (Challis et al., 2020), applying a filter for GC content equal to 0.5 because Tiara (Karlicki et al., 2022) identified contigs with higher GC content as “unknown” or “bacteria.” Furthermore, a filter for HiFi coverage equal to 5,180 was applied as contigs above this level of coverage were identified as organelles by Tiara. Hereafter, we will refer to this assembly as “Hifiasm.”

### 2.3 Hi-C scaffolding

All the Hi-C scaffolders combine Hi-C linkage information with draft genome assemblies to resolve contig orientations.

As a first step, the 3D-DNA algorithm identifies assembly errors where a scaffold’s long-range contact pattern changes unexpectedly. Thereafter, the resulting sequences are anchored, ordered, and oriented via an algorithm based on the contact frequency between pairs of sequences to indicate their proximity in the genome. Finally, contigs and scaffolds that correspond to overlapping regions of the genome are merged (Dudchenko et al., 2017).

SALSA2 builds scaffold graph scoring edges according to a “best buddy” scheme. The scaffolds are then constructed according to a greedy weighted maximum matching. Afterward, SALSA2 performs an iterative step of misjoin detection and correction, which stops naturally when accurate Hi-C links are exhausted (Ghurye et al., 2019).

YaHS is the newest scaffolder that has been developed and compared to previous scaffolders, it proposes a new method for building the contact matrix. The software builds a contact matrix, constructs and prunes a scaffold graph, and outputs the scaffolds (Zhou et al., 2023).

Hi-C reads were trimmed before performing the scaffolding using fastp with the following parameters: -p, --detect_adapter_for_pe, --cut_front, --cut_tail, --cut_window_size 4, and--cut_mean_quality 20.

#### 2.3.1 3D-DNA

Hi-C reads were filtered and aligned to the draft assembly, using Juicer (Durand et al., 2016) with the parameters -p assembly, -s none, and -S early. The fasta file of the contig-level assembly was given as input for both the Juicer flags --g, normally used to input a reference assembly, and --z, to avoid reference biases. Prior to scaffolding, the contig-level assembly was wrapped using the script wrap-fasta-sequence.awk. Finally, the run-asm-pipeline.sh script from 3D-DNA (Dudchenko et al., 2017) was run with the wrapped.fasta file, and the list of Hi-C contacts in .txt format was outputted by Juicer. The scripts used were those provided for a single CPU run.

#### 2.3.2 SALSA2

Hi-C reads were filtered and aligned to the contig-level assembly following the Arima Genomics mapping pipeline (https://github.com/ArimaGenomics/mapping_pipeline). Steps 1A and 1B of the pipeline were modified by adding the flag -M to the bwa mem commands. The resulting bam file was subsequently sorted by read name using samtools sort and converted to a .bed file using bedtools. Finally, the script run_pipeline.py from SALSA2 (Ghurye et al., 2019) was run with the parameters -e GATC and -m yes to perform the scaffolding.

#### 2.3.3 YaHS

The Hi-C reads were filtered and aligned to the contig-level assembly following the same methods described for SALSA2. The output.bam file of the Arima Genomics mapping pipeline was sorted and converted to the .bed format as described above. YaHS was finally run with the parameter -e GATC (Zhou et al., 2023).

### 2.4 AssemblyQC pipeline

We developed assemblyQC, a Bash pipeline designed to perform quality control of assemblies. AssemblyQC combines QUAST, BUSCO, and Merqury and optionally runs Liftoff along with a Python script that produces metrics about gene positioning in the assembly compared to a given reference genome.

QUAST (Mikheenko et al., 2018) is a tool that computes relevant quality metrics useful for evaluating *de novo* assemblies and comparing them against reference sequences. Using the flag -w, assemblyQC runs QUAST-LG, the QUAST extension for evaluating large genomes. In our case, regular QUAST was run.

BUSCO (Manni et al., 2021) evaluates genome assembly quality in terms of gene completeness. To run BUSCO using assemblyQC, it is necessary to previously download the database of the lineage of interest. In our case, this was brassicales_odb10.

Merqury (Rhie et al., 2020) allows reference-free assembly evaluation of accuracy and completeness, comparing the k-mers found in the *de novo* assembly with those present in high-accuracy raw reads. To run Merqury, it is necessary to previously generate a k-mer database using Meryl. Illumina short reads were used to construct the Meryl database. AssemblyQC supports only the case in which one assembly with no hap-mers (i.e., haplotype-specific k-mers) is provided to run Merqury.

Liftoff (Shumate and Salzberg, 2021) is a standalone tool that accurately maps annotations from a reference genome to a target assembly. Reference.fasta and .gff/.gtf files and the .fasta file of the target assembly are needed; the output is a .gff/.gtf file for the target assembly. Assembly QC accepts as input for Liftoff only files in .gff format and optionally filters out organelle annotations before running this program.

AssemblyQC runs Liftoff with the flag --exclude-partial, which is used to exclude partial/low sequence identity mappings (coverage ≥ 0.5 and sequence identity ≥ 0.5) from the output.gff.

liftoff_combine.py is a Python script we developed to output metrics about gene collinearity between the target assembly and the reference genome. We define gene collinearity as the correspondence between the gene position in the target assembly and that in the reference genome. The script takes the output.gff from Liftoff and the reference.gff file as input. The script analyses only gene collinearity; it excludes exons and transcripts from the .gff files. Moreover, the script allows us to set a threshold to evaluate the divergence in intergenic length between pairs of adjacent genes between the target and the reference assemblies: if the divergence is lower than the threshold, the compared genomic annotations are considered coherent (default: 500 bp).

liftoff_combine.py outputs a .txt file per reference chromosome that provides information regarding i) which contigs or scaffolds in the target assembly correspond to the reference chromosome, ii) which orientation contigs or scaffolds in the target assembly have compared to the reference, and iii) whether and in which measure the distance between adjacent genes in the target assembly match the distance between corresponding genes in the reference assembly.

The secondary outputs of this script are a merged assembly-reference.gff for the whole genome, merged.gff files per chromosome, and records of the genes showing divergent gene distances between the reference and the assembly per chromosome.

For this study, we ran assemblyQC, including the Liftoff step. We removed organelle annotations from the reference.gff file and adopted a divergence threshold of 500 bp for liftoff_combine.py. The input k-mer length was determined with the Merqury command best_k.sh.

### 2.5 Evaluation criteria

The evaluation of the two scaffolded assemblies was based on several approaches: 1) contiguity, that is, number of contigs, N50, N90, L50, and L90 and the cumulative length plot, obtained with QUAST; 2) completeness, calculated as i) genome fraction, obtained with QUAST; ii) presence, completeness, and duplication of ortholog genes, calculated by BUSCO comparing the assemblies with the database for Brassicales (brassicales_odb10); iii) k-mer completeness, calculated by Merqury; 3) accuracy, assessed through Merqury consensus quality (QV) and copy number spectrum plots; 4) structural correctness, based on gene collinearity metrics provided by the Liftoff plus liftoff_combine.py method and the Hi-C contact map generated using JuiceBox Assembly Tools (JBAT) (Dudchenko et al., 2018). Furthermore, the three scaffolders were evaluated according to the estimated runtime, determined by examining the timestamps (i.e., wall clock time) of the first file and the most recent files generated by the tools.

### 2.6 Cluster characteristics

The programs in benchmarking were executed on a Linux machine containing two 2.1 GHz, 18-core Intel Xeon E5-2695 (Broadwell) series processors, where each of the cores in these processors supports two hyperthreads enabled by default with 256 GB of memory.

## 3 Results and discussion

### 3.1 YaHS outperforms SALSA2 and 3D-DNA in terms of assembly contiguity

Table 2 shows the QUAST metrics of the scaffolded assemblies compared to the contig-level assemblies Flye and Hifiasm. All the scaffolders were able to align more than 99% of bases to the reference genome of *A. thaliana* (TAIR10.1).

**TABLE 2:** Comparison of the assemblies according to QUAST metrics.

Assembly	Genome fraction (%)	Contigs	N50	N90	L50	L90
Flye	99.097	15	14,864,979	9,471,025	4	8
Flye_3D-DNA	99.068	30	19,600,500	12,160,500	3	6
Flye_SALSA2	99.105	14	15,405,308	9,471,025	4	8
Flye_yahs	99.109	7	24,256,539	19,109,354	3	5
Hifiasm	99.302	5	26,162,003	22,263,862	3	5
Hifiasm_3D-DNA	99.304	342	3,413,500	175,000	8	139
Hifiasm_SALSA2	99.302	8	16,698,585	13,446,829	4	7
Hifiasm_yahs	99.287	4	32,656,027	22,674,124	2	4

SALSA2 and YaHS were able to reduce the number of contigs of the draft assembly, while 3D-DNA increased the number. The best result was achieved by YaHS for both assemblies. YaHS reduced the number of contigs from fifteen to seven in the case of Flye and from five to four in the case of Hifiasm, probably introducing a false joint between two contigs as five chromosomes are expected for *A. thaliana*. Notably, the number of contigs dramatically increased for Hifiasm scaffolded with 3D-DNA, increasing to 342 contigs from the initial five contigs.

Compared with the draft assemblies, all the scaffolders increased the N50, except for Hifiasm scaffolded with 3D-DNA, for which the N50 decreased from the initial ≈26 Mb to ≈3 Mb. Only YaHS increased the N90 for both Flye (from ≈9 Mb to ≈19 Mb) and Hifiasm (from ≈22.3 Mb to ≈22.7 Mb). 3D-DNA increased the N90 only for Flye (from ≈9 Mb to ≈12 Mb), while it strongly decreased the N90 in the case of Hifiasm (from ≈22 Mb to ≈2 Mb). SALSA2 did not produce changes in N90 in the case of Flye, while it decreased N90 for Hifiasm (from ≈22 Mb to ≈13 Mb). YaHS produced the highest N50 and N90 for both assemblies.

Flye L50 was reduced by both 3D-DNA and YaHS, while no L50 changes were produced by SALSA2. Hifiasm L50 was decreased by YaHS. YaHS also achieved the lowest L90 for both assemblies.

The full QUAST report is shown in Supplementary Table S1.

**FIGURE 1:**
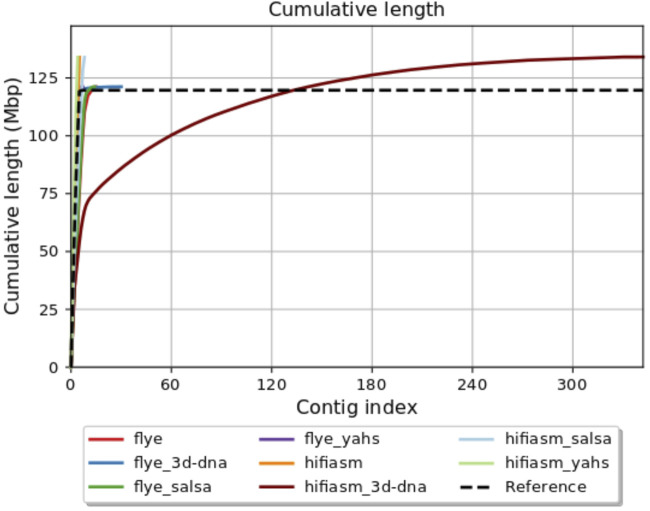
QUAST plot for cumulative length (Mbp).

Figure 1 shows the QUAST plot for cumulative length (Mbp). The Flye assembly was improved by all the scaffolders, with YaHS giving the best results for all the parameters considered. In this case, 3D-DNA slightly increased the fragmentation of the assembly by increasing the number of contigs, but it improved all the other parameters. SALSA2 reduced the number of contigs and increased the N50, keeping the other parameters unchanged.

In conclusion, the Hifiasm assembly showed good metrics at the contig-level. YaHS was the only scaffolder that did not increase assembly fragmentation, but it introduced a false joint, joining two contigs in the same scaffold. SALSA2 and 3D-DNA increased the assembly fragmentation, with the latter representing the most extreme case.

### 3.2 3D-DNA decreased orthologs completeness in the assembly produced with Hifiasm

**FIGURE 2:**
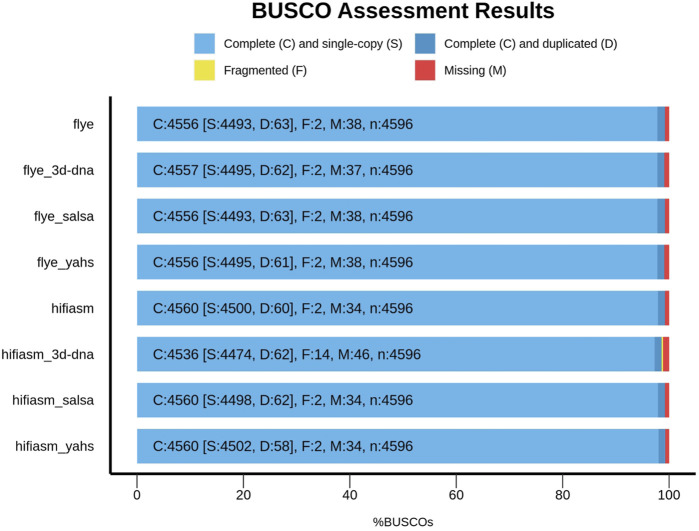
Comparison of the assemblies according to BUSCO metrics.

Figure 2 shows the comparison between Flye and Hifiasm and the relative scaffolded assemblies with respect to the BUSCO metrics.

Compared with those of the contig-level assemblies, the numbers of complete, missing, and fragmented BUSCOs in SALSA2 and YaHS remained unchanged. However, YaHS increased the number of complete and single-copy BUSCOs and decreased the number of duplicated BUSCOs, unlike SALSA2.

3D-DNA behaved differently according to the assembly considered. In the case of Flye, it increased the number of complete BUSCOs, decreased the number of missing BUSCOs, and kept fragmented BUSCOs unaltered compared to those of the contig-level assembly, overall improving the BUSCO metrics. On the other hand, for Hifiasm, 3D-DNA decreased the number of complete BUSCOs and increased the number of missing and fragmented BUSCOs, decreasing the overall metric quality compared to that of the contig-level assembly.

### 3.3 Hi-C scaffolders have little influence on k-mer completeness and consensus quality

Table 3 shows the results obtained for each assembly with Merqury.

**TABLE 3:** Comparison of the assemblies according to Merqury metrics.

Assembly	QV	Error rate	K-mer set used for measuring completeness	Solid k-mers in the assembly	Total solid k-mers in the read set	Completeness (%)
Flye	51.0816	7.795E−06	all	104,943,677	106,416,479	98.616
Flye_3D-DNA	51.0816	7.795E−06	all	104,943,545	106,416,479	98.6159
Flye_SALSA2	51.0816	7.795E−06	all	104,943,677	106,416,479	98.616
Flye_yahs	51.0816	7.795E−06	all	104,943,677	106,416,479	98.616
Hifiasm	60.0914	9.792E−07	all	105,327,621	106,416,479	98.9768
Hifiasm_3D-DNA	60.0911	9.792E−07	all	105,320,145	106,416,479	98.9698
Hifiasm_SALSA2	60.0914	9.792E−07	all	105,327,621	106,416,479	98.9768
Hifiasm_yahs	60.0914	9.792E−07	all	105,327,621	106,416,479	98.9768

The QV values in the scaffolded assemblies compared to those in the contig-level assemblies remained unaltered, except for Hifiasm scaffolded with 3D-DNA, for which the QV decreased from 60.0914 to 60.0911.

All the Flye assemblies showed a level of completeness above 98.6%, and all the Hifiasm assemblies showed completeness >98.9%.

Copy number spectrum plots for each assembly are shown in Supplementary Figure S1. All the assemblies fit the expected copy number spectrum for a haploid genome.

### 3.4 YaHS generates the most structurally correct chromosome-length assemblies

The detailed results of comparing gene positioning in the assemblies and the reference genome TAIR10.1 are reported in Supplementary Tables S2–S6).

Table 4 displays the number of contigs or scaffolds in the target assemblies containing genes mapped to chromosomes 1 to 5 in the reference genome TAIR10.1 according to the Liftoff results.

**TABLE 4:** Number of contigs or scaffolds in the target assemblies containing genes annotated on chromosomes 1 to 5 in the reference genome TAIR10.1 according to the Liftoff results.

Assembly	Chr 1	Chr 2	Chr 3	Chr 4	Chr 5
Flye	2	9	4	4	3
Flye_3D-DNA	5	8	3	2	3
Flye_SALSA2	3	9	4	2	3
Flye_yahs	1	4	2	1	1
Hifiasm	1	1	3	1	1
Hifiasm_3D-DNA	64	36	64	47	73
Hifiasm_SALSA2	2	2	3	1	2
Hifiasm_yahs	1	1	2	1	1

YaHS was the only scaffolder able to reduce the number of scaffolds corresponding to the TAIR10.1 chromosomes according to gene content in comparison to both the contig-level assemblies and for all the chromosomes.

Notably, for Hifiasm, which was initially formed by five contigs (i.e., the expected number of chromosomes), 3D-DNA dramatically increased the number of scaffolds corresponding to the reference chromosomes according to the gene content.

With respect to the orientation of the scaffolds, we considered a contig or scaffold to have a clear orientation with respect to the reference genome when at least 90% of the gene coordinates were in ascending (forward orientation) or descending order (reverse orientation).

With respect to Flye, 3D-DNA provided a clear orientation for all the chromosomes, except for the scaffolds corresponding to chromosome 2, where the proportion of genes in the same orientation as the reference and the proportion of genes in reverse complement orientation were 86.6% and 13.1%, respectively. SALSA2 conserved the orientation of the scaffolds of the Flye contig-level assembly for chromosomes 1 and 3, which were in the same orientation and in reverse complement, respectively, compared to the reference genome. It was not able to provide a clear orientation for the scaffolds corresponding to chromosomes 2, 4, and 5 in TAIR10.1. YaHS was able to provide a clear orientation for all the scaffolds.

In the case of Hifiasm, 3D-DNA was not able to define scaffold orientation for any chromosome, except for the scaffolds corresponding to chromosome 4 of TAIR10.1, which were in reverse complement orientation compared to the reference genome, as the contig-level Hifiasm assembly. SALSA2 showed a clear orientation for the scaffolds corresponding to chromosomes 1, 3, and 5, while the orientation was not clear for chromosomes 2 and 4. YaHS was able to provide a clear orientation for all the scaffolds.

Overall, YaHS was the only scaffolder that decreased the number of scaffolds corresponding to the reference chromosomes for both assemblies. YaHS was also the only scaffolder to provide a clear orientation for all the scaffolds. The collinearity results confirmed that YaHS introduced a false joint in the Hifiasm assembly, merging the scaffolds that corresponded to chromosomes 3 and 4 in TAIR10.1.

The Hifiasm assembly deserves particular attention. This assembly contained only five contigs that mainly corresponded to the chromosomes of TAIR10.1 before scaffolding. The Hifiasm contigs ptg000001l_1 and ptg000002l_1 contained mainly genes corresponding to chromosome 2 and chromosome 4 of TAIR10.1, respectively. However, ptg000001l_1 and ptg000002l_1 also contained seven and two genes, respectively, which were mapped to TAIR10.1 chromosome 3. YaHS partially resolved these misplacement events: only the seven genes that were in contig ptg000001l_1 were placed in the scaffold_4 corresponding to chromosome 2 of TAIR10.1, while the other two genes that were originally mapped to ptg000002l_1 were correctly placed in scaffold_1, corresponding to chromosome 3. SALSA2 did not resolve these misplacements. Due to the high level of fragmentation introduced by 3D-DNA, it was impossible to evaluate how this scaffolder handled these misplacement events (Table 5). Further data are needed to ascertain whether the seven genes of Hifiasm contig ptg000002l_1 represent a misplacement or a true translocation event.

**TABLE 5:** Correspondence between reference chromosome 3, contigs in Hifiasm, and scaffolds in Hifiasm scaffolded with SALSA2 and YaHS for the nine genes involved in an observed misplacement. The detailed correspondence chromosome-contigs and chromosome-scaffolds are reported in Supplementary Tables S2–S6.

Gene ID	Reference chromosome	Hifiasm contig	SALSA2 scaffold	YaHS scaffold
AT3G00650	CP002686.1	ptg000001l_1	scaffold_8	scaffold_4
AT3G41762	CP002686.1	ptg000001l_1	scaffold_8	scaffold_4
AT3G41761	CP002686.1	ptg000001l_1	scaffold_8	scaffold_4
AT3G06345	CP002686.1	ptg000001l_1	scaffold_8	scaffold_4
AT3G41768	CP002686.1	ptg000001l_1	scaffold_8	scaffold_4
AT3G06355	CP002686.1	ptg000001l_1	scaffold_8	scaffold_4
AT3G41979	CP002686.1	ptg000001l_1	scaffold_8	scaffold_4
AT3G00660	CP002686.1	ptg000002l_1	scaffold_2	scaffold_1
AT3G06365	CP002686.1	ptg000002l_1	scaffold_2	scaffold_1

**FIGURE 3:**
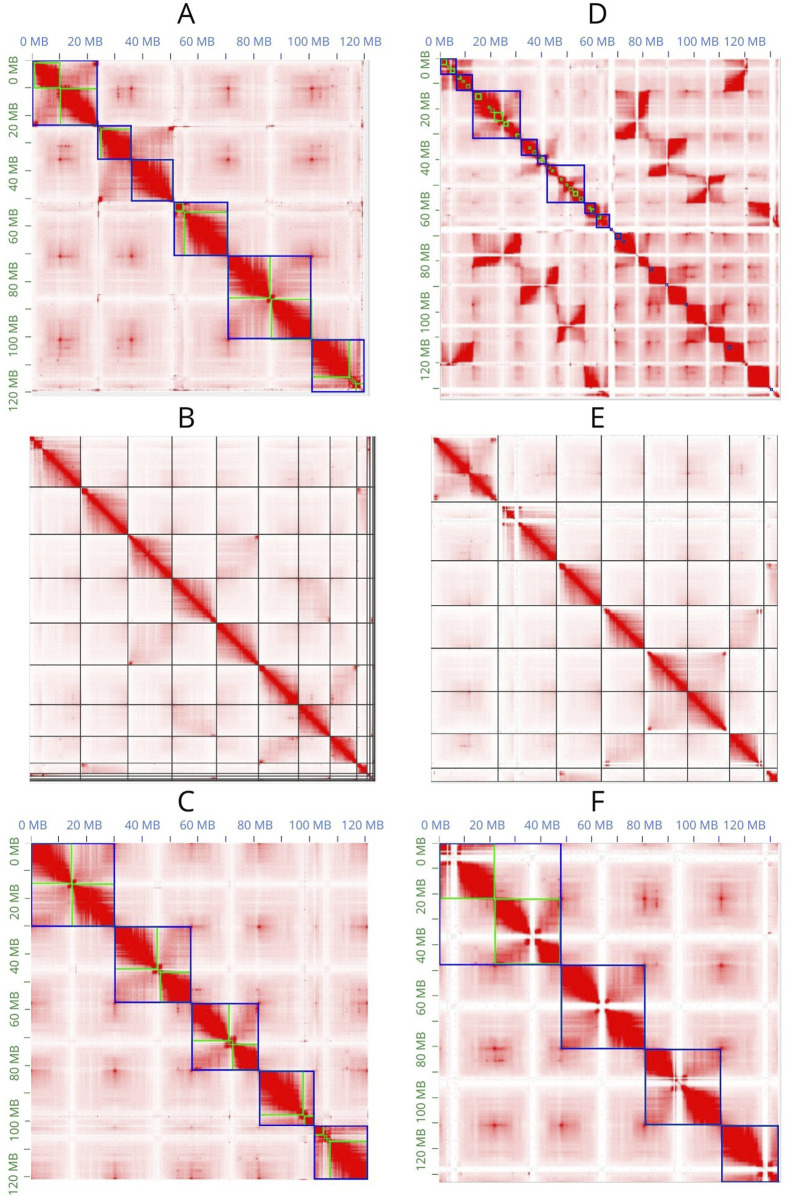
Contact maps obtained with JBAT. **(A–C)** Contact maps of the Flye assembly scaffolded with 3D-DNA, SALSA2, and YaHS, respectively. **(D–F)** A contact map of the Hifiasm assembly scaffolded with 3D-DNA, SALSA2, and YaHS, respectively. In **(A, C, D, F),** the green lines represent the contigs, and the blue lines represent the scaffolds.

JuiceBox Assembly Tools (JBAT) (Dudchenko et al., 2018) allows visualization of the Hi-C contact map and manual curation of the scaffolded assemblies (Figures 3A, C, D, F). SALSA2 provides a method that only allows visualization of the contact map in JBAT but does not allow the highlighting of contigs and scaffolds; therefore, manual curation of the assembly is not possible (Figures 3B, E).

Only YaHS correctly identified the major scaffolds corresponding to the chromosomes in both Flye and Hifiasm (Figures 3C, F). In the latter case, YaHS identified four major scaffolds because two of the chromosome-length scaffolds were joined (Figure 3F). However, the error could be easily detected and corrected with JBAT by manually splitting the two chromosome-length scaffolds.

The Hi-C contact map confirmed that, for both assemblies, 3D-DNA and SALSA2 produced more fragmented assemblies than did YaHS, with the most extreme case observed in Hifiasm scaffolded with 3D-DNA.

### 3.5 YaHS exhibits remarkable speed compared to the other scaffolders

Table 6 displays the estimated runtime (h:mm:ss) for the tools used for the Hi-C pre-processing, specifically Juicer and Arima Genomics mapping pipeline, as well as for the scaffolders, which include 3D-DNA, SALSA2, and YaHS, for the two assemblies considered. As regards the pre-processing, Flye Juicer, used before 3D-DNA, was faster than the Arima Genomics mapping pipeline, used before SALSA2 and YaHS, while we observed little difference between the two pre-processing methods for the Hifiasm assembly.

**TABLE 6:** Estimated runtime (h:mm:ss) for the tools used for the Hi-C pre-processing (i.e., Juicer and Arima Genomics mapping pipeline) and for the scaffolders (i.e., 3D-DNA, SALSA2, and YaHS).

Assembly	Flye			Hifiasm		
Tool	First file last modified	Last file last modified	Estimated runtime	First file last modified	Last file last modified	Estimated runtime
Juicer	11:22:01	13:03:59	1:03:59	15:44:14	17:05:00	1:20:46
Arima Genomics mapping pipeline	12:24:09	13:47:13	1:23:04	14:13:54	15:30:10	1:16:16
3D-DNA	14:11:11	15:26:18	1:15:07	18:51:07	21:07:17	2:16:10
SALSA2	16:32:55	16:52:18	0:19:23	16:22:35	16:37:52	0:15:17
YaHS	15:29:16	15:30:03	0:00:47	16:16:01	16:17:01	0:00:50

In terms of the three scaffolders, YaHS clearly demonstrated an outstanding runtime for both assemblies, requiring less than 1 min to complete the scaffolding. SALSA2 had a decent runtime, taking roughly 20 min for Flye and 15 min for Hifiasm. 3D-DNA was the slowest scaffolder, requiring more than 1 h for scaffolding Flye and more than 2 h for Hifiasm.

## 4 Conclusion

The two assemblies scaffolded with YaHS showed the highest contiguity, completeness, and structural correctness, producing high-quality chromosome-length assemblies for the two investigated cases.

Overall, 3D-DNA performed well with the Flye assembly, but it heavily fragmented the Hifiasm assembly, showing a possible incompatibility between the two tools.

Compared to the other two scaffolders considered, SALSA2 produced intermediate results for both assemblies.

YaHS proved to be the most straightforward software to install and use. Additionally, the developers provided comprehensive documentation to facilitate the implementation of the software in the analysis process. 3D-DNA also has good documentation, but we found that it is more difficult to install and run. With respect to SALSA2, the main flaws are the lack of detailed and rich documentation and the lack of methods for manual curation of the assembly.

YaHS greatly outperformed the other two scaffolders in terms of runtime.

Of note, we did not try different combinations of parameters for the three scaffolders to determine whether they could perform better with other settings.

Our findings align with those presented by the scaffolder developers in their original articles: SALSA2 authors compared their software with 3D-DNA, finding that SALSA2 was producing higher quality results (Ghurye et al., 2019); YaHS authors compared their tool with SALSA2, finding that YaHS can produce higher quality results. To test their tools, both SALSA2 and YaHS developers used real and simulated human datasets. YaHS authors also tested the scaffolders with 15 species belonging to different taxonomic groups, including mammals, insects, fungi, and plants. They demonstrated that YaHS outperformed SALSA2 for most of the species considered, while in a limited number of cases, the results were comparable (Zhou et al., 2023). In the original article, the authors did not compare 3D-DNA with SALSA2 and YaHS due to its earlier development.

Of the three scaffolders that were benchmarked in this study, YaHS was the best performing for most of the parameters considered. Therefore, we conclude that it is the most appropriate scaffolding tool for *de novo* assemblies to date.

## Data Availability

Publicly available datasets were analyzed in this study. These data can be found here: BioProject PRJCA005809 (https://ngdc.cncb.ac.cn/bioproject/browse/PRJCA005809); GitHub assemblyQC repository: https://github.com/LiaOb21/assemblyQC.
