# Supplementary material for: Benchmarking of Hi-C tools for scaffolding plant genomes obtained from PacBio HiFi and ONT reads
Source: Front Bioinform. 2024 Nov 15;4:1462923. doi: 10.3389/fbinf.2024.1462923 (PMC11604747; doi:10.3389/fbinf.2024.1462923)
Supplement: Supplementary file 1 [file DataSheet1.pdf]

Benchmarking of Hi-C tools for scaffolding plant genomes obtained from PacBio HiFi and ONT reads

Lia Obinu [1,2*], Urmi Trivedi [2] and Andrea Porceddu [1]

[1] *Department of Agricultural Sciences, University of Sassari, Sassari, Sardinia, Italy*
[2] *Edinburgh Genomics, The University of Edinburgh, Edinburgh, Scotland, United Kingdom*

Correspondence*:
Lia Obinu
lobinu@uniss.it

SUPPLEMENTARY MATERIALS

Table S1 Full QUAST report.

Assembly	Flye	Flye_3D-DNA	Flye_SALSA2	Flye_yahs	Hifiasm	Hifiasm_3D-DNA	Hifiasm_SALSA2	Hifiasm_yahs
Contigs (≥ 0 bp)	15	30	14	7	5	342	8	4
Contigs (≥ 1000 bp)	15	30	14	7	5	342	8	4
Contigs (≥ 5000 bp)	15	26	14	7	5	331	8	4
Contigs (≥ 10000 bp)	15	25	14	7	5	330	8	4
Contigs (≥ 25000 bp)	15	24	14	7	5	319	8	4
Contigs (≥ 50000 bp)	14	15	13	6	5	266	8	4
Total length (≥ 0 bp)	121168757	121178257	121169757	121169657	133901430	134049930	133902430	133901530
Total length (≥ 1000 bp)	121168757	121178257	121169757	121169657	133901430	134049930	133902430	133901530
Total length (≥ 5000 bp)	121168757	121168012	121169757	121169657	133901430	134034210	133902430	133901530
Total length (≥ 10000 bp)	121168757	121163012	121169757	121169657	133901430	134026210	133902430	133901530
Total length (≥ 25000 bp)	121168757	121152178	121169757	121169657	133901430	133826000	133902430	133901530
Total length (≥ 50000 bp)	121124559	120889192	121125559	121125459	133901430	132386186	133902430	133901530
Contigs	15	30	14	7	5	342	8	4

Continued on next page

Table S1 continued from previous page

Assembly	Flye	Flye_3D-DNA	Flye_SALSA2	Flye_yahs	Hifiasm	Hifiasm_3D-DNA	Hifiasm_SALSA2	Hifiasm_yahs
Largest contig	16316820	30170187	18141904	30360532	32656027	18736000	26162503	48425965
Total length	121168757	121178257	121169757	121169657	133901430	134049930	133902430	133901530
Reference length	119668634	119668634	119668634	119668634	119668634	119668634	119668634	119668634
GC (%)	36.1	36.1	36.1	36.1	36.35	36.35	36.35	36.35
Reference GC (%)	36.06	36.06	36.06	36.06	36.06	36.06	36.06	36.06
N50	14864979	19600500	15405308	24256539	26162003	3413500	16698585	32656027
NG50	14864979	19600500	15405308	24256539	30145414	4379420	17299953	32656027
N90	9471025	12160500	9471025	19109354	22263862	175000	13446829	22674124
NG90	9471025	15336445	9471025	19109354	22674124	300000	16050884	30145414
auN	13070225.3	21496511.8	14140798.4	24933961.5	27403799.5	6054223.9	18680542.8	36103785.1
auNG	13234069	21767690.8	14318180.5	25246712.2	30663072	6781796.2	20902470.3	40397821.1
L50	4	3	4	3	3	8	4	2
LG50	4	3	4	3	2	7	3	2
L90	8	6	8	5	5	139	7	4
LG90	8	5	8	5	4	82	6	3
Misassemblies	389	352	397	387	1665	1244	1689	1660
Misassembled contigs	14	20	13	5	5	25	8	4
Misassembled contigs length	121124559	120786675	121125559	121004459	133901430	74321589	133902430	133901530
Local misassemblies	116	112	109	101	619	374	613	615
Scaffold gap ext. mis.	0	0	0	0	0	2	0	0
Scaffold gap loc. mis.	0	3	1	0	0	0	0	0

Continued on next page

Table S1 continued from previous page

Assembly	Flye	Flye_3D-DNA	Flye_SALSA2	Flye_yahs	Hifiasm	Hifiasm_3D-DNA	Hifiasm_SALSA2	Hifiasm_yahs
Unaligned mis. contigs	1	2	1	2	0	29	0	0
Unaligned contigs	0 + 15 part	0 + 14 part	0 + 14 part	0 + 7 part	0 + 5 part	8 + 60 part	0 + 8 part	0 + 4 part
Unaligned length	1185893	1230774	1142845	1166251	11515295	11681283	11510549	11532188
Genome fraction (%)	99.097	99.068	99.105	99.109	99.302	99.304	99.302	99.287
Duplication ratio	1.011	1.011	1.011	1.011	1.027	1.027	1.027	1.027
N's per 100 kbp	0	7.84	0.83	0.74	0	110.78	0.75	0.07
Mismatches per 100 kbp	47.74	48.16	48.38	48.23	69.55	70.56	69.75	69.18
Indels per 100 kbp	7.93	8.09	8.03	7.98	7.97	7.92	7.97	7.91
Genomic features	707323 + 132 part	707001 + 164 part	707323 + 132 part	707326 + 129 part	708153 + 145 part	706427 + 1757 part	708159 + 145 part	708159 + 145 part
Largest alignment	9640198	9639526	9640198	9639526	9639574	2451962	9640246	9639574
Total aligned length	119695370	119690055	119740121	119721272	121877392	121849805	121903928	121892201
NA50	3956242	3982976	3956242	3956242	3601484	425000	3601484	3601484
NGA50	3956242	3982976	3956242	3956242	3956256	500000	3956256	3956256
NA90	841493	841493	841493	841493	4404	4199	4404	4404
NGA90	841965	882551	841965	841965	842402	81261	842402	842402
auNA	4310743.1	4325041.6	4309990.1	4312285.1	3899311.8	604328.3	3899704.3	3899858.8
auNGA	4364781.1	4379602.1	4364054.6	4366374.8	4363076.7	676954.1	4363548.5	4363692
LA50	10	10	10	10	12	83	12	12
LGA50	10	10	10	10	10	67	10	10
LA90	37	36	37	37	317	870	316	313

Continued on next page

Table S1 continued from previous page

Assembly	Flye	Flye_3D-DNA	Flye_SALSA2	Flye_yahs	Hifiasm	Hifiasm_3D-DNA	Hifiasm_SALSA2	Hifiasm_yahs
LGA90	36	35	36	36	36	284	36	36

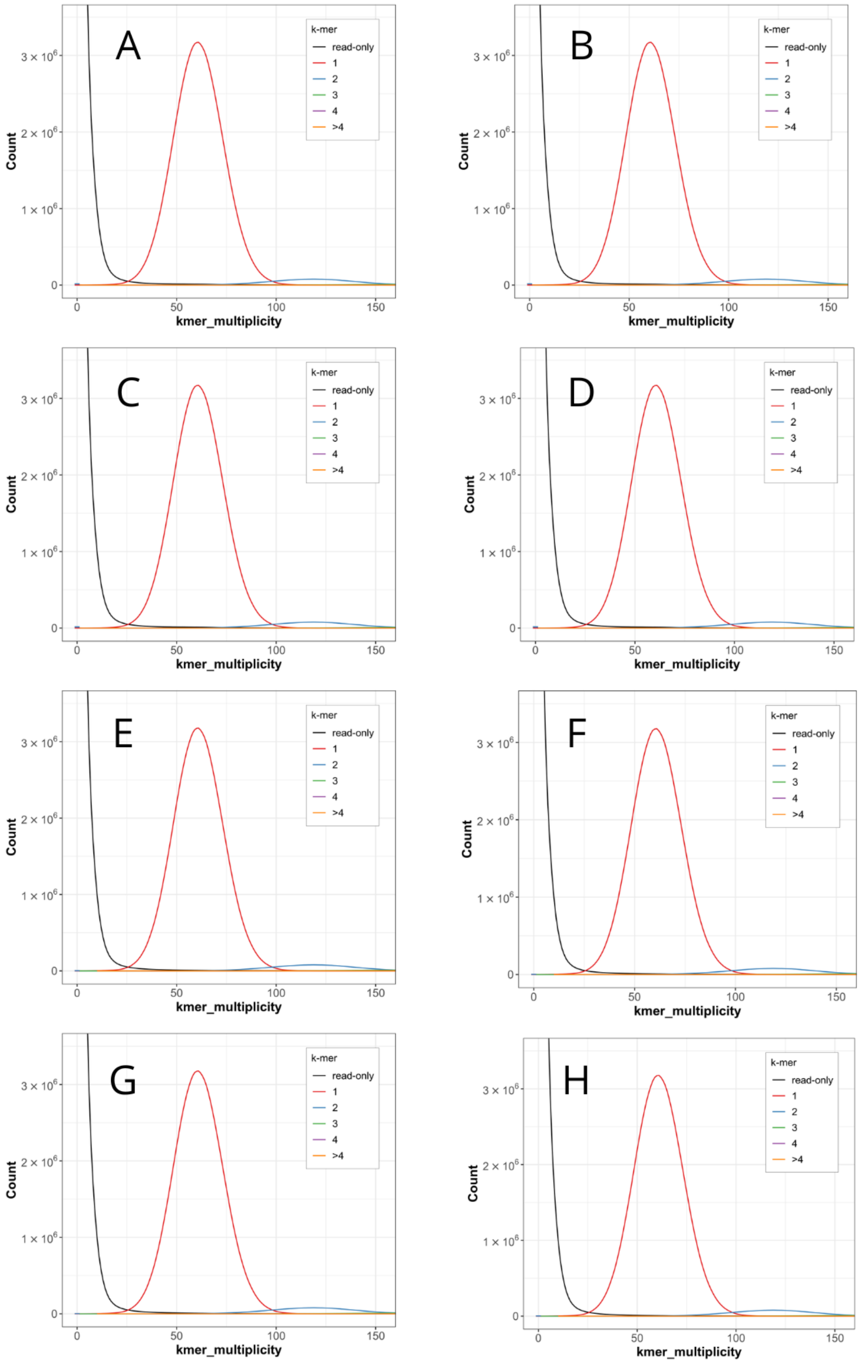

Figure S1. Copy number spectrum plots obtained with Merqury.
A) Flye contig-level assembly, B) Flye scaffolded with 3D-DNA, C) Flye scaffolded with SALSA2, D) Flye scaffolded with YaHS, E) Hifiasm contig-level assembly, F) Hifiasm scaffolded with 3D-DNA, G) Hifiasm scaffolded with SALSA2, H) Hifiasm scaffolded with YaHS.

Table S2 Liftoff_combine.py results for contigs or scaffolds containing genes mapping to chromosome 1 of the reference genome TAIR10.1.

Assembly	Contigs or scaffolds corresponding to chromosome 1	Gene count	Genes showing divergent gene distance	Reverse complement	Genes (%) in ascending order	Genes (%) in descending order	Total number of genes
Flye	contig_21_1	4356	24	no	99.500000	0.200000	8740.000000
	contig_5_1	4384	31				
Flye_3D-DNA	HiC_scaffold_15	1	1	yes	6.700000	93.100000	8740.000000
	HiC_scaffold_16	1	1				
	HiC_scaffold_2	2	2				
	HiC_scaffold_5	8735	8393				
	HiC_scaffold_9	1	1				
Flye_SALSA2	scaffold_2	1	1	no	99.500000	0.200000	8740.000000
	scaffold_3	4355	23				
	scaffold_5	4384	31				
Flye_yahs	scaffold_1	8740	54	no	99.500000	0.200000	8740.000000
Hifiasm	ptg000007l_1	8738	56	no	99.500000	0.200000	8738.000000
Hifiasm_3D-DNA	HiC_scaffold_14	6	1	undetermined	54.300000	45.400000	8734.000000
	HiC_scaffold_15	9	1				
	HiC_scaffold_20	6	1				
	HiC_scaffold_22	914	2				
	HiC_scaffold_23	88	1				
	HiC_scaffold_24	16	1				
	HiC_scaffold_25	289	1				
	HiC_scaffold_26	176	2				
	HiC_scaffold_27	81	1				
	HiC_scaffold_28	55	1				
	HiC_scaffold_29	78	1				
	HiC_scaffold_3	4785	4083				
	HiC_scaffold_30	3	1				

Continued on next page

Table S2 continued from previous page

Assembly	Contigs or scaffolds corresponding to chromosome 1	Gene count	Genes showing divergent gene distance	Reverse complement	Genes (%) in ascending order	Genes (%) in descending order	Total number of genes
	HiC_scaffold_31	97	2				
	HiC_scaffold_32	166	2				
	HiC_scaffold_33	34	1				
	HiC_scaffold_34	18	1				
	HiC_scaffold_35	86	2				
	HiC_scaffold_36	13	7				
	HiC_scaffold_37	37	1				
	HiC_scaffold_38	17	1				
	HiC_scaffold_39	65	2				
	HiC_scaffold_40	53	1				
	HiC_scaffold_41	31	1				
	HiC_scaffold_42	27	1				
	HiC_scaffold_43	86	1				
	HiC_scaffold_44	86	1				
	HiC_scaffold_45	15	1				
	HiC_scaffold_46	22	1				
	HiC_scaffold_47	19	1				
	HiC_scaffold_48	34	1				
	HiC_scaffold_49	21	1				
	HiC_scaffold_50	7	1				
	HiC_scaffold_51	9	1				
	HiC_scaffold_68	40	1				
	HiC_scaffold_69	38	1				
	HiC_scaffold_70	68	1				
	HiC_scaffold_71	27	1				
	HiC_scaffold_72	43	1				
	HiC_scaffold_73	39	1				
	HiC_scaffold_74	14	1				

Continued on next page

Table S2 continued from previous page

Assembly	Contigs or scaffolds corresponding to chromosome 1	Gene count	Genes showing divergent gene distance	Reverse complement	Genes (%) in ascending order	Genes (%) in descending order	Total number of genes
	HiC_scaffold_75	12	1				
	HiC_scaffold_76	8	1				
	HiC_scaffold_77	86	1				
	HiC_scaffold_78	56	1				
	HiC_scaffold_79	3	1				
	HiC_scaffold_80	7	1				
	HiC_scaffold_81	16	1				
	HiC_scaffold_82	40	1				
	HiC_scaffold_83	33	1				
	HiC_scaffold_84	9	1				
	HiC_scaffold_85	59	1				
	HiC_scaffold_86	12	1				
	HiC_scaffold_87	8	1				
	HiC_scaffold_88	13	1				
	HiC_scaffold_89	10	1				
	HiC_scaffold_90	101	2				
	HiC_scaffold_91	36	1				
	HiC_scaffold_92	87	1				
	HiC_scaffold_93	44	1				
	HiC_scaffold_94	57	1				
	HiC_scaffold_95	130	1				
	HiC_scaffold_96	127	1				
	HiC_scaffold_97	62	1				
Hifiasm_SALSA2	scaffold_5	4356	24	no	99.500000	0.200000	8738.000000
	scaffold_6	4382	32				
Hifiasm_yahs	scaffold_2	8738	55	no	99.500000	0.200000	8738.000000

Table S3 Liftoff_combine.py results for contigs or scaffolds containing genes mapping to chromosome 2 of the reference genome TAIR10.1.

Assembly	Contigs or scaffolds corresponding to chromosome 2	Gene count	Genes showing divergent gene distance	Reverse complement	Genes (%) in ascending order	Genes (%) in descending order	Total number of genes
Flye	contig_16_1	1	1	yes	6.100000	93.700000	5190.000000
	contig_1_1	724	696				
	contig_21_1	1	1				
	contig_27_1	1	1				
	contig_31_1	2	2				
	contig_4_1	4458	4294				
	contig_5_1	1	1				
	contig_66_1	1	1				
	contig_6_1	1	1				
Flye_3D-DNA	HiC_scaffold_1	2	2	undetermined	86.600000	13.100000	5192.000000
	HiC_scaffold_2	3	3				
	HiC_scaffold_22	22	19				
	HiC_scaffold_23	4	4				
	HiC_scaffold_24	3	2				
	HiC_scaffold_3	1	1				
	HiC_scaffold_4	5155	680				
	HiC_scaffold_5	2	2				
Flye_SALSA2	scaffold_13	2	2	undetermined	86.600000	13.100000	5191.000000
	scaffold_2	4459	9				
	scaffold_3	1	1				
	scaffold_4	1	1				
	scaffold_5	1	1				
	scaffold_6	1	1				
	scaffold_7	1	1				
	scaffold_8	1	1				

Continued on next page

Table S3 continued from previous page

Assembly	Contigs or scaffolds corresponding to chromosome 2	Gene count	Genes showing divergent gene distance	Reverse complement	Genes (%) in ascending order	Genes (%) in descending order	Total number of genes
	scaffold_9	724	696				
Flye_yahs	scaffold_1	2	2	yes	6.000000	93.800000	5192.000000
	scaffold_2	2	2				
	scaffold_3	4	4				
	scaffold_4	5184	4990				
Hifiasm	ptg000011l_1	5260	5064	yes	5.900000	93.900000	5260.000000
Hifiasm_3D-DNA	HiC_scaffold_1	809	29	undetermined	44.600000	55.100000	5259.000000
	HiC_scaffold_2	1664	325				
	HiC_scaffold_240	175	166				
	HiC_scaffold_241	204	193				
	HiC_scaffold_242	231	225				
	HiC_scaffold_243	447	429				
	HiC_scaffold_244	20	20				
	HiC_scaffold_245	55	52				
	HiC_scaffold_246	84	83				
	HiC_scaffold_247	85	82				
	HiC_scaffold_248	94	90				
	HiC_scaffold_249	25	24				
	HiC_scaffold_250	37	35				
	HiC_scaffold_251	156	142				
	HiC_scaffold_252	96	89				
	HiC_scaffold_253	192	185				
	HiC_scaffold_254	71	68				
	HiC_scaffold_255	27	26				
	HiC_scaffold_256	46	45				
	HiC_scaffold_257	69	67				
	HiC_scaffold_258	22	22				

Continued on next page

Table S3 continued from previous page

Assembly	Contigs or scaffolds corresponding to chromosome 2	Gene count	Genes showing divergent gene distance	Reverse complement	Genes (%) in ascending order	Genes (%) in descending order	Total number of genes
	HiC_scaffold_259	6	6				
	HiC_scaffold_260	83	81				
	HiC_scaffold_261	6	6				
	HiC_scaffold_262	276	262				
	HiC_scaffold_263	36	36				
	HiC_scaffold_264	125	120				
	HiC_scaffold_265	10	9				
	HiC_scaffold_266	7	7				
	HiC_scaffold_268	3	3				
	HiC_scaffold_269	3	3				
	HiC_scaffold_279	63	60				
	HiC_scaffold_280	24	22				
	HiC_scaffold_282	1	1				
	HiC_scaffold_283	4	4				
	HiC_scaffold_342	3	3				
Hifiasm_SALSA2	scaffold_3	4461	11	undetermined	85.500000	14.300000	5261.000000
	scaffold_8	800	768				
Hifiasm_yahs	scaffold_4	5260	5064	yes	5.900000	93.900000	5261.000000

Table S4 Liftoff_combine.py results for contigs or scaffolds containing genes mapping to chromosome 3 of the reference genome TAIR10.1.

Assembly	Contigs or scaffolds corresponding to chromosome 3	Gene count	Genes showing divergent gene distance	Reverse complement	Genes (%) in ascending order	Genes (%) in descending order	Total number of genes
Flye	contig_11_1	10	5	yes	5.900000	93.900000	6543.000000
	contig_27_1	2650	2565				
	contig_31_1	8	8				
	contig_6_1	3875	3748				
Flye_3D-DNA	HiC_scaffold_1	6533	14	no	99.600000	0.100000	6543.000000
	HiC_scaffold_26	3	2				
	HiC_scaffold_6	7	5				
Flye_SALSA2	scaffold_1	10	7	yes	5.900000	93.900000	6543.000000
	scaffold_12	8	8				
	scaffold_6	3875	3748				
	scaffold_8	2650	2565				
Flye_yahs	scaffold_3	6533	14	no	99.700000	0.100000	6543.000000
	scaffold_5	10	5				
Hifiasm	ptg000001l_1	7	7	no	99.600000	0.100000	6542.000000
	ptg000002l_1	2	2				
	ptg000003l_1	6533	14				
Hifiasm_3D-DNA	HiC_scaffold_17	5	1	undetermined	76.081970	23.673345	6540.000000
	HiC_scaffold_173	251	1				
	HiC_scaffold_174	177	1				
	HiC_scaffold_175	94	1				
	HiC_scaffold_176	177	1				
	HiC_scaffold_177	118	1				
	HiC_scaffold_178	150	1				
	HiC_scaffold_179	103	1				
	HiC_scaffold_18	7	1				

Continued on next page

Table S4 continued from previous page

Assembly	Contigs or scaffolds corresponding to chromosome 3	Gene count	Genes showing divergent gene distance	Reverse complement	Genes (%) in ascending order	Genes (%) in descending order	Total number of genes
	HiC_scaffold_180	180	1				
	HiC_scaffold_181	145	3				
	HiC_scaffold_182	200	1				
	HiC_scaffold_183	32	1				
	HiC_scaffold_184	149	1				
	HiC_scaffold_185	81	1				
	HiC_scaffold_186	17	1				
	HiC_scaffold_187	176	1				
	HiC_scaffold_188	181	1				
	HiC_scaffold_189	7	1				
	HiC_scaffold_19	3	1				
	HiC_scaffold_190	19	1				
	HiC_scaffold_191	6	1				
	HiC_scaffold_192	23	1				
	HiC_scaffold_193	30	1				
	HiC_scaffold_194	61	1				
	HiC_scaffold_195	6	1				
	HiC_scaffold_196	48	1				
	HiC_scaffold_197	26	1				
	HiC_scaffold_198	31	1				
	HiC_scaffold_199	40	1				
	HiC_scaffold_200	66	1				
	HiC_scaffold_201	3	1				
	HiC_scaffold_202	2	1				
	HiC_scaffold_213	1	1				
	HiC_scaffold_214	1	1				
	HiC_scaffold_215	24	2				
	HiC_scaffold_216	2	1				

Continued on next page

Table S4 continued from previous page

Assembly	Contigs or scaffolds corresponding to chromosome 3	Gene count	Genes showing divergent gene distance	Reverse complement	Genes (%) in ascending order	Genes (%) in descending order	Total number of genes
	HiC_scaffold_217	14	1				
	HiC_scaffold_218	46	1				
	HiC_scaffold_219	8	1				
	HiC_scaffold_220	7	1				
	HiC_scaffold_221	91	1				
	HiC_scaffold_222	77	2				
	HiC_scaffold_223	26	1				
	HiC_scaffold_224	69	1				
	HiC_scaffold_225	159	1				
	HiC_scaffold_226	138	1				
	HiC_scaffold_227	108	1				
	HiC_scaffold_228	215	1				
	HiC_scaffold_229	63	1				
	HiC_scaffold_230	63	1				
	HiC_scaffold_231	224	1				
	HiC_scaffold_232	82	1				
	HiC_scaffold_233	277	1				
	HiC_scaffold_234	7	1				
	HiC_scaffold_235	22	1				
	HiC_scaffold_236	43	1				
	HiC_scaffold_237	66	1				
	HiC_scaffold_238	59	1				
	HiC_scaffold_239	125	1				
	HiC_scaffold_284	7	6				
	HiC_scaffold_342	1	1				
	HiC_scaffold_6	1900	1561				
	HiC_scaffold_8	1	1				
Hifiasm_SALSA2	scaffold_1	6533	6321	yes	5.900000	93.900000	6542.000000

Continued on next page

Table S4 continued from previous page

Assembly	Contigs or scaffolds corresponding to chromosome 3	Gene count	Genes showing divergent gene distance	Reverse complement	Genes (%) in ascending order	Genes (%) in descending order	Total number of genes
	scaffold_2	2	2				
	scaffold_8	7	6				
Hifiasm_yahs	scaffold_1	6535	16	no	99.600000	0.100000	6542.000000
	scaffold_4	7	6				

Table S5 Liftoff_combine.py results for contigs or scaffolds containing genes mapping to chromosome 4 of the reference genome TAIR10.1.

Assembly	Contigs or scaffolds corresponding to chromosome 4	Gene count	Genes showing divergent gene distance	Reverse complement	Genes (%) in ascending order	Genes (%) in descending order	Total number of genes
Flye	contig_11_1	737	4	no	98.800000	1.000000	4998.000000
	contig_14_1	17	3				
	contig_18_1	4200	21				
	contig_67_1	44	42				
Flye_3D-DNA	HiC_scaffold_20	1	1	yes	5.500000	94.200000	4998.000000
	HiC_scaffold_6	4997	4814				
Flye_SALSA2	scaffold_1	4954	746	undetermined	84.900000	14.900000	4998.000000
	scaffold_10	44	42				
Flye_yahs	scaffold_5	4998	31	no	99.600000	0.100000	4998.000000
Hifiasm	ptg000002l_1	5000	4818	yes	5.500000	94.200000	5000.000000
Hifiasm_3D-DNA	HiC_scaffold_285	293	278	yes	9.700000	90.000000	4999.000000
	HiC_scaffold_286	9	9				

Continued on next page

Table S5 continued from previous page

Assembly	Contigs or scaffolds corresponding to chromosome 4	Gene count	Genes showing divergent gene distance	Reverse complement	Genes (%) in ascending order	Genes (%) in descending order	Total number of genes
	HiC_scaffold_287	75	72				
	HiC_scaffold_288	7	7				
	HiC_scaffold_289	223	213				
	HiC_scaffold_290	103	100				
	HiC_scaffold_291	155	148				
	HiC_scaffold_292	120	116				
	HiC_scaffold_293	241	238				
	HiC_scaffold_294	98	95				
	HiC_scaffold_295	213	208				
	HiC_scaffold_296	283	275				
	HiC_scaffold_297	17	17				
	HiC_scaffold_298	135	124				
	HiC_scaffold_299	82	79				
	HiC_scaffold_300	34	34				
	HiC_scaffold_301	21	19				
	HiC_scaffold_302	30	29				
	HiC_scaffold_303	7	7				
	HiC_scaffold_304	13	13				
	HiC_scaffold_305	21	21				
	HiC_scaffold_306	68	67				
	HiC_scaffold_307	16	15				
	HiC_scaffold_308	55	53				
	HiC_scaffold_309	36	35				
	HiC_scaffold_310	32	31				
	HiC_scaffold_311	146	140				
	HiC_scaffold_312	28	27				
	HiC_scaffold_313	103	102				
	HiC_scaffold_314	95	95				

Continued on next page

Table S5 continued from previous page

Assembly	Contigs or scaffolds corresponding to chromosome 4	Gene count	Genes showing divergent gene distance	Reverse complement	Genes (%) in ascending order	Genes (%) in descending order	Total number of genes
	HiC_scaffold_315	42	41				
	HiC_scaffold_326	3	3				
	HiC_scaffold_328	12	12				
	HiC_scaffold_329	15	15				
	HiC_scaffold_330	14	13				
	HiC_scaffold_331	7	7				
	HiC_scaffold_332	10	10				
	HiC_scaffold_333	7	6				
	HiC_scaffold_334	6	5				
	HiC_scaffold_336	54	54				
	HiC_scaffold_337	16	16				
	HiC_scaffold_338	8	7				
	HiC_scaffold_339	31	31				
	HiC_scaffold_340	33	31				
	HiC_scaffold_341	98	95				
	HiC_scaffold_7	1252	1068				
	HiC_scaffold_8	632	579				
Hifiams_SALSA2	scaffold_2	5000	750	undetermined	85.600000	14.100000	5000.000000
Hifiasm_yahs	scaffold_1	5000	33	no	99.600000	0.200000	5000.000000

Table S6 Liftoff_combine.py results for contigs or scaffolds containing genes mapping to chromosome 5 of the reference genome TAIR10.1.

Assembly	Contigs or scaffolds corresponding to chromosome 5	Gene count	Genes showing divergent gene distance	Reverse complement	Genes (%) in ascending order	Genes (%) in descending order	Total number of genes
Flye	contig_13_1	11	1	undetermined	46.7	53.1	7449
	contig_16_1	4148	4034				
	contig_66_1	3290	7				
Flye_3D-DNA	HiC_scaffold_2	3301	3198	yes	5	94.9	7449
	HiC_scaffold_21	1	1				
	HiC_scaffold_3	4147	4033				
Flye_SALSA2	scaffold_11	11	1	undetermined	46.7	53.1	7449
	scaffold_4	4148	4034				
	scaffold_7	3290	7				
Flye_yahs	scaffold_2	7449	7222	yes	5.1	94.7	7449
Hifiasm	ptg000006l_1	7447	37	no	99.7	0.1	7447
Hifiasm_3D-DNA	HiC_scaffold_1	1	1	undetermined	69	30.8	7443
	HiC_scaffold_100	545	2				
	HiC_scaffold_101	80	1				
	HiC_scaffold_102	23	1				
	HiC_scaffold_103	39	1				
	HiC_scaffold_104	109	1				
	HiC_scaffold_105	105	2				
	HiC_scaffold_106	110	1				
	HiC_scaffold_107	43	1				
	HiC_scaffold_108	183	1				
	HiC_scaffold_109	146	1				
	HiC_scaffold_110	62	1				
	HiC_scaffold_111	39	1				
	HiC_scaffold_112	71	2				

Continued on next page

Table S6 continued from previous page

Assembly	Contigs or scaffolds corresponding to chromosome 5	Gene count	Genes showing divergent gene distance	Reverse complement	Genes (%) in ascending order	Genes (%) in descending order	Total number of genes
	HiC_scaffold_113	140	1				
	HiC_scaffold_114	12	1				
	HiC_scaffold_115	68	1				
	HiC_scaffold_116	172	1				
	HiC_scaffold_117	13	1				
	HiC_scaffold_118	14	1				
	HiC_scaffold_119	16	1				
	HiC_scaffold_120	73	1				
	HiC_scaffold_121	14	1				
	HiC_scaffold_122	20	1				
	HiC_scaffold_123	20	1				
	HiC_scaffold_124	20	1				
	HiC_scaffold_125	17	1				
	HiC_scaffold_126	10	1				
	HiC_scaffold_134	7	1				
	HiC_scaffold_135	1	1				
	HiC_scaffold_136	3	1				
	HiC_scaffold_137	2	1				
	HiC_scaffold_138	3	1				
	HiC_scaffold_139	7	1				
	HiC_scaffold_140	53	1				
	HiC_scaffold_141	1	1				
	HiC_scaffold_142	23	1				
	HiC_scaffold_143	15	1				
	HiC_scaffold_144	28	1				
	HiC_scaffold_145	70	1				
	HiC_scaffold_146	49	1				
	HiC_scaffold_147	89	1				

Continued on next page

Table S6 continued from previous page

Assembly	Contigs or scaffolds corresponding to chromosome 5	Gene count	Genes showing divergent gene distance	Reverse complement	Genes (%) in ascending order	Genes (%) in descending order	Total number of genes
	HiC_scaffold_148	9	1				
	HiC_scaffold_149	25	1				
	HiC_scaffold_150	30	1				
	HiC_scaffold_151	17	1				
	HiC_scaffold_152	22	1				
	HiC_scaffold_153	35	1				
	HiC_scaffold_154	65	1				
	HiC_scaffold_155	32	2				
	HiC_scaffold_156	82	1				
	HiC_scaffold_157	97	1				
	HiC_scaffold_158	63	1				
	HiC_scaffold_159	110	1				
	HiC_scaffold_160	52	1				
	HiC_scaffold_161	11	1				
	HiC_scaffold_162	67	1				
	HiC_scaffold_163	158	1				
	HiC_scaffold_164	69	1				
	HiC_scaffold_165	233	1				
	HiC_scaffold_166	96	1				
	HiC_scaffold_167	33	1				
	HiC_scaffold_168	140	1				
	HiC_scaffold_169	26	1				
	HiC_scaffold_170	204	1				
	HiC_scaffold_171	124	2				
	HiC_scaffold_172	268	21				
	HiC_scaffold_2	22	1404				
	HiC_scaffold_4	1682	36				
	HiC_scaffold_5	48	871				

Continued on next page

Table S6 continued from previous page

Assembly	Contigs or scaffolds corresponding to chromosome 5	Gene count	Genes showing divergent gene distance	Reverse complement	Genes (%) in ascending order	Genes (%) in descending order	Total number of genes
	HiC_scaffold_6	1000	1				
	HiC_scaffold_98	33	1				
	HiC_scaffold_99	74					
Hifiasm_SALSA2	scaffold_4	4146	27	no	99.7	0.1	7447
	scaffold_7	3301	9				
Hifiasm_yahs	scaffold_3	7447	37	no	99.7	0.1	7447
